# Broiler Farms and Carcasses Are an Important Reservoir of Multi-Drug Resistant *Escherichia coli* in Ecuador

**DOI:** 10.3389/fvets.2020.547843

**Published:** 2020-11-25

**Authors:** David Ortega-Paredes, Sofía de Janon, Fernando Villavicencio, Katherine Jaramillo Ruales, Kenny De La Torre, José E. Villacís, Jaap A. Wagenaar, Jorge Matheu, Camila Bravo-Vallejo, Esteban Fernández-Moreira, Christian Vinueza-Burgos

**Affiliations:** ^1^Unidad de Investigación de Enfermedades Transmitidas por Alimentos y Resistencia a los Antimicrobianos (UNIETAR), Facultad de Medicina Veterinaria y Zootecnia, Universidad Central del Ecuador, Quito, Ecuador; ^2^Centro de Referencia Nacional de Resistencia a los Antimicrobianos, Instituto Nacional de Investigación en Salud Pública “Leopoldo Izquieta Pérez”, Quito, Ecuador; ^3^Facultad de Medicina, Pontificia Universidad Católica del Ecuador, Quito, Ecuador; ^4^Wageningen Bioveterinary Research, Lelystad, Netherlands; ^5^Department of Infectious Diseases and Immunology, Faculty of Veterinary Medicine, Utrecht University, Utrecht, Netherlands; ^6^Department of Food Safety and Zoonoses, World Health Organization, Geneva, Switzerland; ^7^Hospital General del Sur Quito-Instituto Ecuatoriano de Seguridad Social (IESS), Quito, Ecuador; ^8^Carrera de Medicina, Facultad de Ciencias de la Salud, Universidad de las Américas, Quito, Ecuador

**Keywords:** AmpC beta-lactamases, broiler farms, broiler carcasses, *E. coli*, extended-spectrum beta-lactamase (ESBL), human, mcr-1

## Abstract

Antimicrobial resistance (AMR) is a major health threat for public and animal health in the twenty-first century. In Ecuador, antibiotics have been used by the poultry industry for decades resulting in the presence of multi-drug resistant (MDR) bacteria in the poultry meat production chain, with the consequent risk for public health. This study evaluated the prevalence of ESBL/AmpC and *mcr* genes in third-generation cephalosporin-resistant *Escherichia coli* (3GC-R *E. coli*) isolated from broiler farms (animal component), broiler carcasses (food component), and human enteritis (human component) in Quito-Ecuador. Samples were collected weekly from November 2017 to November 2018. For the animal, food, and human components, 133, 335, and 302 samples were analyzed, respectively. Profiles of antimicrobial resistance were analyzed by an automated microdilution system. Resistance genes were studied by PCR and Sanger sequencing. From all samples, 122 (91.7%), 258 (77%), and 146 (48.3%) samples were positive for 3GC-R *E. coli* in the animal, food, and human components, respectively. Most of the isolates (472/526, 89.7%) presented MDR phenotypes. The ESBL *bla*_CTX-*M-*55_, *bla*_CTX-*M-*3_, *bla*_CTX-*M-*15_, *bla*_CTX-*M-*65_, *bla*_CTX-*M-*27_, and *bla*_CTX-*M-*14_ were the most prevalent ESBL genes while *bla*_CMY-2_ was the only AmpC detected gene. The *mcr*-1 gene was found in 20 (16.4%), 26 (10.1%), and 3 (2.1%) of isolates from animal, food, and human components, respectively. The implication of poultry products in the prevalence of ESBL/AmpC and *mcr* genes in 3GC-R must be considered in the surveillance of antimicrobial resistance.

## Introduction

The World Health Organization recognizes antimicrobial resistance (AMR) as a major health threat in the 21st century (1). A global projection predicts that the increase of deaths linked to AMR will develop from 700,000 in 2016 to 10 million deaths per year in 2050 while 100 trillion USD could be lost by 2050 (2). In this scenario, the use of antibiotics in food animal production is one of the most important issues contributing to the AMR crisis. In fact, over 50% of antibiotic production is used by the meat industry and an increase of 50% in antibiotic usage for farming is estimated by 2030. Moreover, up to a 160% increase in antibiotic usage in food animals is expected in Latin American countries in absence of changes (3).

Poultry production is an important sector for the study of AMR because of the common usage of antibiotics in this industry. Additionally, it is expected that poultry will be the main animal production industry by 2025 (4). This issue is especially significant in developing countries where antimicrobials are not only used to treat infections but also prophylactically and as growth promoters (5). In Ecuador, poultry products are the most important source of animal protein with a per capita consumption of poultry meat of 30.4 kg/year (6).

In Ecuador, commonly used antibiotics in poultry production include quinolones, fosfomycin, and colistin, which are listed by WHO as critically important antimicrobials for human medicine, with quinolones and colistin even highest prioritized (7). This practice has promoted the dissemination of multi-drug resistant (MDR) bacteria, principally extended-spectrum β-lactamases and AmpC-producing (3GC-R) *Escherichia coli*, which is commonly studied as a sentinel organism to understand the epidemiology of AMR (8). In Ecuador, colistin was banned for use in food animals in 2019. However, *mcr* genes have been recently identified in *E. coli* isolated from animals, humans, and the environment (9–11).

A relationship between 3GC-R *E. coli* isolated from poultry products and humans has been suggested previously (12–16). In Ecuador, Vinueza-Burgos et al. described a high prevalence of 3GC-R *E. coli* and the presence of *mcr*-1 in poultry farms (17). However, there is no data about the prevalence of 3GC-R and colistin-resistant *E. coli* in broiler carcasses and humans in this location. Therefore, this study aimed to evaluate the state of 3GC-R and *mcr* genes in *E. coli* in broiler farms, chicken carcasses at retail level and human stool samples in Ecuador.

## Materials and Methods

### Study Design and Sampling

Samples for *E. coli* isolation were collected weekly from November 2017 to November 2018. Under local legislation, ethical approval was not required for collecting chicken caeca and carcasses during sampling. The participants for the human component were informed about the objective of the study; the participation was voluntary (all volunteers provided a written consent) and all personal information was anonymized. This project was approved by the committee of bioethics from the National Institute of Public Health “Leopoldo Izquieta Pérez” (Protocol ID: CEISH-INSPI-005). The sample distribution for each component considered local characteristics (location of farms in the zone of Quito, distribution of retail shops in the city, and location of healthcare centers).

The sample size for the animal component was calculated considering the number of industrial farms close to Quito, the number of batches that these farms produce in 1 year, and the prevalence of 3GC-R *E. coli* in poultry farms previously reported in this zone (17). For the food and human components, the minimum sample size was calculated considering an infinite population and at a 0.9 of confidence.

For the animal component,133 flocks from 69 farms close to Quito were sampled during the study period. For every sampled flock, 25 caeca from 25 chicken were randomly collected at the slaughterhouse and transported to the laboratory in an icebox within 2 h. At the laboratory, a sample pool of 25 g. was obtained and homogenized by hand as previously described (17).

For the food component, 335 carcasses were collected in three kinds of markets as follows: 125 samples from supermarkets, 126 samples from small shops, and 84 samples from open markets. Sampling places were distributed in both northern and southern areas of Quito. A sampling of chicken carcasses was performed alternately between the north and south of the city. Each sample consisted of one carcass collected in its original bag and transported to the laboratory in an icebox within 2 h. At the laboratory, 25 g of breast skin of every carcass were aseptically collected for bacteriological analysis.

For the human component, stool samples were collected in two health care centers located in the urban periphery of Quito. The inclusion criteria of the patients from whom samples were taken were: individuals with two or more episodes of diarrhea or vomiting in the last 24 h. Stool samples were transported to the laboratory in an icebox within 2 h and 25 g of feces were collected for bacteriological analysis.

### Isolation and Identification of Cefotaxime-Resistant *E. coli*

All samples were homogenized with 225 ml of buffered peptone water (BPW; Difco, BD, Sparks, MD) and incubated at 37°C for 18–24 h. A loopful of each sample was streaked onto chromogenic Tryptone Bile X-Glucuronide (TBX) agar (BioRad, Hercules, California, USA) supplemented with cefotaxime (3 mg/l). Positive plates were considered when at least one typical colony could be selected (when available, two colonies were selected for further analysis) and confirmed to be *E. coli* using Triple Sugar Iron (TSI) agar (Difco, BD, Detroit, USA) and by PCR as described elsewhere (18). From the TSI medium, one loopful was suspended in 300 μl of sterile water and used to extract DNA by the boiling method. Another loopful was used to subculture the isolate in trypticase soy broth (TSB) (Difco, BD, Detroit, USA) and stored with glycerol (60%) at −80°C.

### Antimicrobial Susceptibility Testing and Resistance Genes Screening

Resistance profiles to antibiotics commonly tested for *Enterobacteraceae* were obtained for all isolates using the Vitek® 2 system with AST-N271 cards (BioMérieux, Marcy-l'Étoile, France). The following antibiotics were tested: ampicillin, ampicillin + sulbactam, cephalothin, cefuroxime, ceftriaxone, cefotaxime, ceftazidime, cefepime, ertapenem, meropenem, amikacin, gentamicin, ciprofloxacin, norfloxacin, fosfomycin, nitrofurantoin, and trimethoprim + sulfamethoxazole. The MIC for colistin was tested in isolates positive for *mcr* genes by PCR using BD Phoenix™ M50 with NMIC/ID 94 panel (Becton Dickinson, Nueva Jersey, USA). Antimicrobial resistance phenotypes were obtained following the manufacturer's instructions. *E. coli* ATCC 25922 was used as a quality control strain. Results were evaluated using the clinical breakpoints recommended by CLSI (19). Isolates resistant to at least 3 antibiotic classes were considered as MDR. The *mcr* 1 to 5, *bla*_CTX-*M*_, *bla*_TEM_, *bla*_SHV_, and *bla*_CMY_ genes were tested by PCR in all isolates as previously described (17, 20). Obtained amplicons were sequenced at Macrogen (Seoul-South Korea). Sequences were analyzed using Genious Prime software with the ResFinder database (21).

### Statistical Analysis

Significate differences of the prevalence of 3GC-R *E. coli* within and among each component were calculated using a χ^2^ test. This statistical test was also used to assess differences in antibiotics resistance rates and the presence of resistance genes variants as well as determining differences among farms, types of markets, and location of health care centers (*p* < 0.05). The 95% confidence intervals (CI_95%_) for the prevalence of 3GC-R *E. coli* was obtained by Binomial “exact” calculation. All tests were carried out in RStudio V.1.2.

## Results

### Isolation of *Escherichia coli* Resistant to Cefotaxime

A total of 526 samples were positive for 3GC-R *E. coli*. The highest prevalence was identified in the animal component (91.7%; CI_95%_: 90.8–92.7), followed by the food component (77%; CI_95%_: 76.3–77.8) and the human component (48.3%; CI_95%_: 47.9–48.8). χ^2^ test identified significant differences among components (*p* < 0.05). However, no significant differences were observed within farms, types of shops, and locations of health centers (*p* > 0.05).

### Antimicrobial Susceptibility Testing

All the isolates were resistant to ampicillin, cephalothin, cefuroxime, ceftriaxone, cefotaxime, and trimethoprim + sulfamethoxazole. High resistance rates (more than 80%), were registered for cefepime and ceftazidime in the three components. Resistance to ciprofloxacin and norfloxacin ranged from 70 to 80% in the animal and food components and were significantly higher than rates in the human component, where half of the isolates were resistant. AMR to the combination β-lactam + β-lactamase inhibitor (Ampicillin + Sulbactam) was around 55% in the three components.

Resistance to fosfomycin, nitrofurantoin, and gentamicin showed significant differences between the three components ranging from 30 to 50% in animal and food components and from 24 to 30% in the human component, while for cefepime the resistance rate was higher in humans (*p* < 0.05). Five isolates (two from the animal, one from food, and two from human components) were resistance to carbapenems (ertapenem and/or meropenem). None of the isolates were resistant to Amikacin (Table 1). Additionally, 89.7% of the isolates (472/526), presented MDR patterns with three up to seven groups of antibiotics. The most frequent pattern included resistance to β-lactams, fluoroquinolones, and folate pathway inhibitors (Table 2, Supplementary File 1). Distribution of MIC values for every tested antibiotic in each component shown in Supplementary File 4.

**Table 1:** Antibiotic resistance rates in each component.

**Antibiotic**	**Animal component** ***n* = 122**	**Food component** ***n* = 258**	**Human component** ***n* = 146**	**Total** ***n* = 526**
Ceftazidime	111 (91%)	244 (94,6%)	142 (97,3%)	497 (94,5%)
Cefepime	100 (82%)	225 (87,2%)	133 (91,1%)	458 (87,1%)
*Ciprofloxacin	*92 (75,4%)	*209 (81%)	86 (58,9%)	387 (73,6%)
*Norfloxacin	*86 (70,5%)	*187 (72,5%)	76 (52,1%)	349 (66,3%)
Ampicillin + Sulbactam	65 (53,3%)	147 (57%)	83 (56,8%)	295 (56,1%)
*Fosfomycin	*62 (50,8%)	*127 (49,2%)	43 (29,5%)	232 (44,1%)
*Nitrofurantoin	44 (36,1%)	*105 (40,7%)	40 (27,4%)	189 (35,9%)
*Gentamicin	36 (29,5%)	*96 (37,2%)	35 (24%)	167 (31,7%)
Ertapenem	2 (1,6%)	1 (0,4%)	2 (1,4%)	5 (1%)
Meropenem	1 (0,8%)	0	0	1 (0,2%)

* *Antibiotics in which significant differences (p < 0.05) were identified*.

*Antibiotics with resistance rates or 100% and 0% were not included in this table*.

**Table 2:** Antimicrobial resistance patterns by antibiotic family.

**N^*o*^ antibiotic families**	**Pattern**	**Animal component**	**Food component**	**Human component**	**Total**
7	BEAFPNS		1		1
6	BAFPNS	10	17	8	35
5	BAFNS	9	14	6	29
5	BAFPS	8	25	5	38
5	BFPNS	10	29	3	42
5	BEAFS			1	1
5	BEFPS	1			1
5	BEPNS	1			1
5	BAPNS		1		1
4	BFNS	13	39	8	60
4	BFPS	14	31	13	58
4	BAFS	7	25	12	44
4	BAPS		7	1	8
4	BPNS	1		1	2
4	BEFS			1	1
3	BPS	17	16	12	45
3	BAS	2	6	2	10
3	BFS	20	28	29	77
3	BNS		4	14	18
2	BS	9	15	30	54
Total		122	258	146	526

*B, β-lactams from first line to third generation cephalosporin; E, carbapenems; A, Aminoglycosides; F, fluoroquinolones; P, Phosphonates; N, Nitrofurans; S, Folate pathway inhibitor*.

### Resistance Genes

Group 1 of *bla*_CTX-*M*_ genes was the most prevalent family of ESBL genes, followed by group 9, group 2, and group 8. The allele *bla*_CTX-*M-*55_, belonging to group 1, was identified as the most prevalent variant in the three components, followed by *bla*_CTX-*M-*3_ in animal and food components and *bla*_CTX-*M-*15_ in the human component. Among *bla*_CTX-*M*_ group 9, the most frequent allele in the three components was *bla*_CTX-*M-*65_ followed by *bla*_CTX-*M-*27_ (animal and food components) and *bla*_CTX-*M-*14_ (human component). The *bla*_CTX-*M-*2_ gene was present in the animal and food components, while the *bla*_CTX-*M-*8_ gene was detected in animal and human components. Moreover, ESBL variants of *bla*_SHV_ and broad-spectrum β-lactamases *bla*_TEM_ were frequently identified in the three components. Additionally, *bla*_CTX-*M-*1_, *bla*_CTX-*M-*17_, and *bla*_CTX-*M-*2_ were registered only in poultry isolates, *bla*_CTX-*M-*8_ was identified in food and human components, and *bla*_CTX-*M-*15_, *bla*_CTX-*M-*14_, and *bla*_CTX-*M-*27_ were identified only in the human component. Finally, only the AmpC gene *bla*_CMY-2_ was detected in the samples from the three components (Table 3, Supplementary File 2).

**Table 3:** Prevalence of ESBL/AmpC genes in the cefotaxime-resistant *Escherichia coli* isolates.

**Gen family**	**Allele**	**Animal component**	**Food component**	**Human component**	**Total**
*bla_*CTX*_ group 1*	*nd*		3		3
	*bla_*CTX-M-1*_*		1		1
	*bla_*CTX-M-123*_*	1	3		4
	*bla_*CTX-M-15*_*		2	26	28
	*bla_*CTX-M-27*_*			1	1
	*bla_*CTX-M-3*_*	6	17	13	36
	*bla_*CTX-M-55*_*	50	109	35	194
*bla_*CTX*_ group 9*	*bla_*CTX-M-14*_*			6	6
	*bla_*CTX-M-17*_*	1			1
	*bla_*CTX-M-27*_*			19	19
	*bla_*CTX-M-65*_*	27	56	21	104
*bla_*CTX*_ group 2*	*bla_*CTX-M-2*_*	9	11		20
*bla_*CTX*_ group 8*	*bla_*CTX-M-8*_*		1	2	3
*bla_*CTX*_ group nd*	–	1		2	3
*CMY*	*nd*		4		4
	*bla_*CMY-2*_*	19	38	21	78
TEM*	*nd*		2	3	5
	*bla_*TEM-1*_*	19	74	42	135
	*bla_*TEM-166*_*			1	1
	*bla_*TEM-176*_*		2		2
	*bla_*TEM-1*b**_*	1	4	11	16
*SHV*	*nd*		1	2	3
	*bla_*SHV-12*_*	3	4	11	18
	*bla_*SHV-2*a**_*	2		1	3
	*bla_*SHV-5*_*	3	1	6	10
*nd*	–	8	15	7	30
Total		122	258	146	526

* *The bla_TEM_ alleles identified are not ESBL*.

*nd, not determined*.

The *mcr*-1 gene was found in 16.4% (20/122), 10.1% (26/258), and 2.1% (3/146) of isolates from the animal, food, and human components, respectively. Colistin resistant isolates showed MIC values from 2 to 4 μg/ml. In these isolates, the allele *bla*_CTX-*M-*55_ was dominant in animal and food components, followed by *bla*_CTX-*M-*65_, *bla*_CTX-*M-*2_, and *bla*_CTX-*M-*3_. Additionally, *bla*_CMY-2_ and *bla*_SHV-5_ genes were detected in these components while the alleles *bla*_CTX-*M-*15_ and *bla*_CTX-*M-*55_ were the only ones detected *mcr*-1 positive isolates from humans (Supplementary File 3).

## Discussion

Studies of 3GC-R *E. coli* in poultry production remain scarce in the Andean region of South America (15, 20). Additionally, only a few of these studies have evaluated the prevalence of *mcr* genes in 3GC-R *E. coli* from poultry (17). To the best of our knowledge, this issue has not been evaluated with a multi-component approach in this region.

The high prevalence of 3GC-R *E. coli* in poultry showed in this study is in concordance with a previous report (17). These issues could be related to the rapid dissemination of 3GC-R mediated by horizontal transfer in broiler farms (22). On the other hand, the prevalence in the food component was significantly lower than in the animal component. This fact has been observed in Brazil, where 3GC-R *E. coli* was isolated from 54.2 and 29.2% of samples coming from animals and chicken carcasses, respectively (23). Besides, around 50% of the human stool samples were positive for 3GC-R *E. coli*. Prevalence of 3GC-R *E. coli* in healthy carriers has been reported in the Netherlands, Japan, India, Libya, and Sweden ranging from >5 to 19% (24). Moreover, *E. coli* ESBL has been registered in 18.8% of pediatric patients with diarrhea (25). Our results suggest that the poultry environment in Ecuador is a reservoir of 3GC-R *E. coli*.

Carbapenem resistance mediated by *bla*_KPC-2_, *bla*_KPC-3_, *bla*_OXA-48_, *bla*_NDM-1_, and *bla*_VIM-2_ has been reported in *Enterobacteriaceae* in South American countries in human isolates (26). In Ecuador, *bla*_KPC-2_ is the most prevalent carbapenemase gene detected in *E. coli* in hospitals (27, 28), and recently it was detected in an urban river in *Citrobacter freundii* (29). However, there are no reports of carbapenemases from poultry in our region (carbapenems are not used for poultry production). In our study, the isolates resistant to imipenem/meropenem from the three components were negative for the *bla*_KPC_ gene by PCR testing (data not shown). Therefore, this resistance could be related to not tested carbapenemase genes or to a combination of ESBL enzymes and porin loss (30).

In this study, resistance to fluoroquinolones was significantly higher in isolates originated in poultry (up to 80%) than in isolates originated in the human component (55%). These data are concordant with the common use of quinolones in the poultry industry in Ecuador (31). Additionally, ciprofloxacin is commonly used as an empiric treatment of community-acquired *E. coli* infections in humans. These practices could explain the high resistance rates observed for fluoroquinolones which has also been reported in other countries of the region (32). Even though resistance to fluoroquinolones could be explained by chromosomal point mutations (33), quinolone resistance mediated by mobile resistance genes (*qnr*) has been frequently associated with ESBL production in Enterobacteriaceae (34) suggesting that the co-transference of these genes is a common event.

Additionally, all the isolates in this study were resistant to trimethoprim + sulfamethoxazole. This finding could be explained by the presence of class 1 integrons. These site-specific recombination systems typically have a *sul* gene in their 3′CS end, and frequently present aminoglycoside, quinolone, and β-lactam resistance genes in the variable region. These features promote the selection and evolution of these genetic platforms in mixed antibiotic pressure environments (35), which could explain our findings. However, a genetic analysis to test those elements is necessary to confirm this hypothesis.

The resistance rates to nitrofurantoin reported in this study are higher than the ones reported previously in Ecuador and Colombia (36). However, this antibiotic is not used in the poultry industry. The mechanism of resistance and the reason for the increase of resistance rates reported in this study remain unknown. Our results also showed that half of the isolates from poultry and about 30% of human isolates were resistant to fosfomycin. In South American countries resistance to fosfomycin in *E. coli* isolated from human infections has been reported ranging from 2 to 3% (37, 38). On the other hand, a study carried out in Brazil reported a lower prevalence of this resistance in poultry. There are no other reports of fosfomycin resistance in poultry in neighboring countries, but a close relation of Enterobacteria isolated from poultry between Peru and Ecuador has been described before, proposing the hypothesis of a common epidemiology of these bacteria in the Andes region (30, 31). Nitrofurantoin and fosfomycin are antibiotics prescribed for the treatment of infections caused by MDR and extremely resistant enterobacteria (39, 40). Our findings highlight the urgency of a better regulation of the usage of these antibiotics in Ecuador.

Finally, the aminoglycoside amikacin is not used in poultry production and is restricted to complicated infections in humans (41), so the susceptibility of all isolates to amikacin is not surprising.

In this study, *bla*_CTX-*M-*55_ was the most prevalent allele of the *bla*_CTX-*M*_ group, followed by *bla*_CTX-*M-*65_ and *bla*_CTX-*M-*2_ in the poultry components. These results show a change in the prevalence of *bla*_CTX-*M*_ genes compared with the report a previous report where *bla*_CTX-*M-*65_, *bla*_CTX-*M-*55_, and *bla*_CTX-*M-*3_ were the most prevalent alleles in poultry isolates (17). These outcomes differ with reports in other Latino-American countries as Colombia, where the most dominant allele was *bla*_CMY-2_ (42), and Brazil where *bla*_CTX-*M-*8_ and *bla*_CTX-*M-*2_ are the most important alleles in poultry (37, 38, 43, 44). Additionally, in the human component *bla*_CTX-*M-*55_, *bla*_CTX-*M-*15_, and *bla*_CTX-*M-*65_ were the most prevalent alleles. Based on clinical evidence, the change of the dominance of *bla*_CTX-*M-*15_ to *bla*_CTX-*M-*55_ in humans in Ecuador was already hypostatized in 2016 by Zurita et al. (45).

The presence of component-specific alleles of *bla*_CTX-*M*_ genes also suggests the existence of specific reservoirs. Moreover, it has been hypothesized that the ecological characteristics were animal husbandry is carried out in the region (e.g., altitude, lack of seasons, etc.) could be related to specificities in the epidemiology of these genetic determinants (15, 29). Additionally, it should be noted that the methods used to screen the presence of 3GC-R *E. coli* could give biased information. For example, the use of selective media containing ceftazidime could contribute to recover isolates carrying ceftazidimases with low affinity to cefotaxime; principally alleles of *bla*_CTX-*M*_, *bla*_TEM_, *bla*_SHV_, *bla*_PER_, *bla*_VEB_, *bla*_TLA_, and *bla*_GES/IBC_ (46). On the other hand, media supplemented with cefoxitin could be used for the proper isolation of AmpC β-lactamase-producing *E. coli*.

In Ecuador the *mcr* genes have been reported before in *E. coli* isolated from humans and animals (9–11, 15); but, to the best of our knowledge, this is the first time that genetic determinants for colistin resistance are studied in a multiple-component frame in our region. From all *mcr* genes tested in this study, only the *mcr*-1 gene was detected. This gene was reported in a previous study with a lower prevalence (17) which could indicate that the prevalence of *mcr*-1 in the poultry production has increased. On the other hand, the prevalence of *mcr*-1 in the human component remains low. This relation has been emphasized before, pointing out the animal production origin of this gene (47). Although the use of colistin as a growth promotor has been recently banned in Ecuador, the former intensive use of this antibiotic in the poultry production could be seen as the main trigger for the prevalence observed in the animal component. Our results show that poultry production is an important reservoir of 3GC-R *E. coli* that harbors the *mcr*-1 gene.

It is worth to mention that, as some of the tested antibiotics in this study are not used in poultry production (e.g., cephalosporins and nitrofurantoin), the resistance of *E. coli* to these drugs could be mediated by co-selection events (selection of mixed AR under the pressure of single agent) (48). Genomic elements as conjugating plasmids, insertion sequences, and integrons could play a main role in the dissemination and accumulation of AMR determinants in the poultry production environment. Therefore, co-selection and co-resistance processes should be considered when implementing strategies for AMR control. Besides, environmental factors as contamination of upstream rivers with antibiotics and resistant bacteria should be considered in this analysis (49).

Poultry production has been recognized as an important environment for the evolution of AMR worldwide (50, 51). Indeed, new configurations of resistance genes have been described from poultry production. Important examples are the colocation of *mcr*-1 and *mcr*-3 in plasmids (52), and the emergence of *E. coli* strains co-carrying ESBL and *fos*A3 genes (38). These findings suggest that the antibiotic pressure in poultry production promotes the active recombination and selection of MDR genotypes. Therefore, the presence of strains with new combinations of genetic determinants is a real possibility that should be further studied. In fact, the MDR patterns found in this study suggest the presence of multiple resistance mechanisms that deserve a deeper analysis at the genomic level.

### Concluding Remarks

The high prevalence of 3GC-R *E. coli* reported in this study is worrisome in terms of public health and highlights the need for health policies to prevent the increase of AR in the country.

Additionally, the high prevalence of 3GC- R *E. coli* registered in our study poses a risk of transmission to humans via the food chain. However, the implication of poultry products in the epidemiology of 3GC-R *E. coli* needs further research since other sources for the human acquisition of these bacteria should be considered.

To the best of our knowledge, this study shows for the first time, data on 3GC-R *E. coli* with a multi-component approach in Latin America. We stress the importance of MDR phenotypes and genetic determinants that are spreading rapidly worldwide.

## Data Availability Statement

The original contributions presented in the study are included in the article/Supplementary Material, further inquiries can be directed to the corresponding author/s.

## Author Contributions

DO-P: sampling, interpreted the results, and wrote the manuscript. SJ and KDLT: sampling, interpreted the results, and database management. FV and JV: designed the study, sampling, and performed the experiments in human isolates. JW and JM: designed the study. EF-M: designed the study, provided the critical input, and interpreted the results. CB-V and KR: performed the colistin phenotyping experiments. CV-B: designed the study, provided the critical input, interpreted the results, and wrote the manuscript. KR and KDLT: performed critical laboratory analysis and data analysis. All authors contributed to the article and approved the submitted version.

## Conflict of Interest

The authors declare that the research was conducted in the absence of any commercial or financial relationships that could be construed as a potential conflict of interest.

## Supplementary Material

Supplementary File 1 Antimicrobial resistance patterns by Antibiotic.

Supplementary File 2 Genotyping of the isolates.

Supplementary File 3 Phenotyping and genotyping of *mcr*-1 positive strains.

Supplementary File 4 Distribution of MIC values.
