# Supplementary material for: Broiler Farms and Carcasses Are an Important Reservoir of Multi-Drug Resistant Escherichia coli in Ecuador
Source: Front Vet Sci. 2020 Nov 25;7:547843. doi: 10.3389/fvets.2020.547843 (PMC7724036; doi:10.3389/fvets.2020.547843)
Supplement: Supplementary File 1 — Antimicrobial resistance patterns by Antibiotic. [file Table_1.DOCX]

**Supplementary 1. Antimicrobial resistance patterns by Antibiotic.**

|  |  |  |  |  |  |
| --- | --- | --- | --- | --- | --- |
| **Antibiotics number** | **Resistance**  **pattern** | | **Animal**  **Component** | **Food**  **component** | **Human**  **component** |
| 15 | ABDHCJLREGPOFNT | | 1 |  | 1 |
| 14 | ABDHCJLRGPOFNT | 5 | 8 | 6 | 19 |
| 13 | ABDHCJLGPOFNT | 1 |  |  | 1 |
| 13 | ABDHCJLREGPOT | 1 |  |  | 1 |
| 13 | ABDHCJLREMFNT |  |  | 1 | 1 |
| 13 | ABDHCJLRGPFNT | 1 | 1 |  | 2 |
| 13 | ABDHCJLRGPOFT | 4 | 11 | 2 | 17 |
| 13 | ABDHCJLRGPONT | 4 | 5 | 3 | 12 |
| 13 | ABDHCJLRPOFNT | 1 | 10 | 3 | 14 |
| 13 | ADHCJLRGPOFNT | 1 | 8 | 4 | 13 |
| 12 | ABDHCJLGPOFT |  |  | 2 | 2 |
| 12 | ABDHCJLGPONT |  | 1 |  | 1 |
| 12 | ABDHCJLPOFNT |  | 1 |  | 1 |
| 12 | ABDHCJLRGFNT |  | 1 |  | 1 |
| 12 | ABDHCJLRGPFT |  | 3 |  | 3 |
| 12 | ABDHCJLRGPOT | 4 | 15 | 3 | 22 |
| 12 | ABDHCJLRPFNT |  | 1 | 1 | 2 |
| 12 | ABDHCJLRPOFT | 6 | 12 | 5 | 23 |
| 12 | ABDHCJLRPONT | 2 | 7 | 1 | 10 |
| 12 | ADHCJLRGPOFT | 1 | 10 | 4 | 15 |
| 12 | ADHCJLRGPONT | 2 | 5 | 5 | 12 |
| 12 | ADHCJLRPOFNT | 1 | 16 | 3 | 20 |
| 11 | ABDHCJLEPFT |  |  | 1 | 1 |
| 11 | ABDHCJLGPNT |  | 1 |  | 1 |
| 11 | ABDHCJLGPOT |  | 1 |  | 1 |
| 11 | ABDHCJLPOFT |  | 1 | 1 | 2 |
| 11 | ABDHCJLPONT | 2 | 6 | 3 | 11 |
| 11 | ABDHCJLRFNT | 1 |  |  | 1 |
| 11 | ABDHCJLRGFT |  | 5 |  | 5 |
| 11 | ABDHCJLRGPT |  | 1 |  | 1 |
| 11 | ABDHCJLRPFT | 1 | 5 | 1 | 7 |
| 11 | ABDHCJLRPOT | 10 | 11 | 5 | 26 |
| 11 | ABDHCLGPOFT |  | 1 |  | 1 |
| 11 | ABDHCLGPONT |  | 2 | 1 | 3 |
| 11 | ABDHCLPOFNT |  |  | 2 | 2 |
| 11 | ABHCJLRPONT | 1 |  |  | 1 |
| 11 | ADHCJLREPOT | 1 |  |  | 1 |
| 11 | ADHCJLRGPOT | 7 | 8 | 4 | 19 |
| 11 | ADHCJLRPFNT |  | 1 |  | 1 |
| 11 | ADHCJLRPOFT | 4 | 12 | 7 | 23 |
| 11 | ADHCJLRPONT | 2 | 17 | 6 | 25 |
| 11 | ADHCLRPOFNT | 1 |  |  | 1 |
| 10 | ABDHCJLGFT | 1 |  |  | 1 |
| 10 | ABDHCJLPOT | 1 | 3 | 3 | 7 |
| 10 | ABDHCJLRFT | 6 | 5 | 6 | 17 |
| 10 | ABDHCJLRGT | 1 | 5 | 2 | 8 |
| 10 | ABDHCJLRNT | 5 |  |  | 5 |
| 10 | ABDHCJLRPT | 2 | 3 |  | 5 |
| 10 | ABDHCLPFNT |  |  | 1 | 1 |
| 10 | ABDHCLPONT | 1 | 6 | 3 | 10 |
| 10 | ADHCJLRFNT |  |  | 1 | 1 |
| 10 | ADHCJLRGFT |  | 2 |  | 2 |
| 10 | ADHCJLRGPT | 1 |  |  | 1 |
| 10 | ADHCJLRPFT | 2 | 1 |  | 3 |
| 10 | ADHCJLRPNT |  | 2 |  | 2 |
| 10 | ADHCJLRPOT | 13 | 7 | 9 | 29 |
| 9 | ABDHCJLFT | 1 |  |  | 1 |
| 9 | ABDHCJLGT | 1 | 1 |  | 2 |
| 9 | ABDHCJLNT | 1 | 2 |  | 3 |
| 9 | ABDHCJLPT |  |  | 1 | 1 |
| 9 | ABDHCJLRT | 15 | 4 | 4 | 23 |
| 9 | ABDHCLPNT |  | 1 |  | 1 |
| 9 | ABDHCLPOT |  | 2 |  | 2 |
| 9 | ADHCJLRFT | 5 | 11 | 10 | 26 |
| 9 | ADHCJLRNT | 8 | 1 |  | 9 |
| 9 | ADHCJLRPT | 3 | 2 | 1 | 6 |
| 9 | ADHCLRPOT |  |  | 1 | 1 |
| 8 | ABDHCJLT | 2 | 2 | 1 | 5 |
| 8 | ABDHCLFT |  |  | 1 | 1 |
| 8 | ABDHCLNT |  | 1 |  | 1 |
| 8 | ADHCJLRT | 11 | 8 | 2 | 21 |
| 7 | ABDHCLT | 1 | 1 | 2 | 4 |
| 3 | ABT | 1 |  |  | 1 |
|  | **Total** | **122** | **258** | **146** | **526** |

(A) ampicillin, (B) ampicillin + sulbactam, (D) cephalothin, (H) cefuroxime, (C) cefotaxime, (J) ceftazidime, (L) ceftriaxone, (R) cefepime, (E) ertapenem, (M) meropenem, (K) amikacin, (G) gentamicin, (P) ciprofloxacin, (O) norfloxacin, (F) fosfomycin, (N) nitrofurantoin, (T) trimetoprim + sulfamethoxazole.
