# Supplementary material for: Broiler Farms and Carcasses Are an Important Reservoir of Multi-Drug Resistant Escherichia coli in Ecuador
Source: Front Vet Sci. 2020 Nov 25;7:547843. doi: 10.3389/fvets.2020.547843 (PMC7724036; doi:10.3389/fvets.2020.547843)

Comparison of the distribution of MIC values in the three components. Red lines indicate the breakpoints for each antimicrobial.

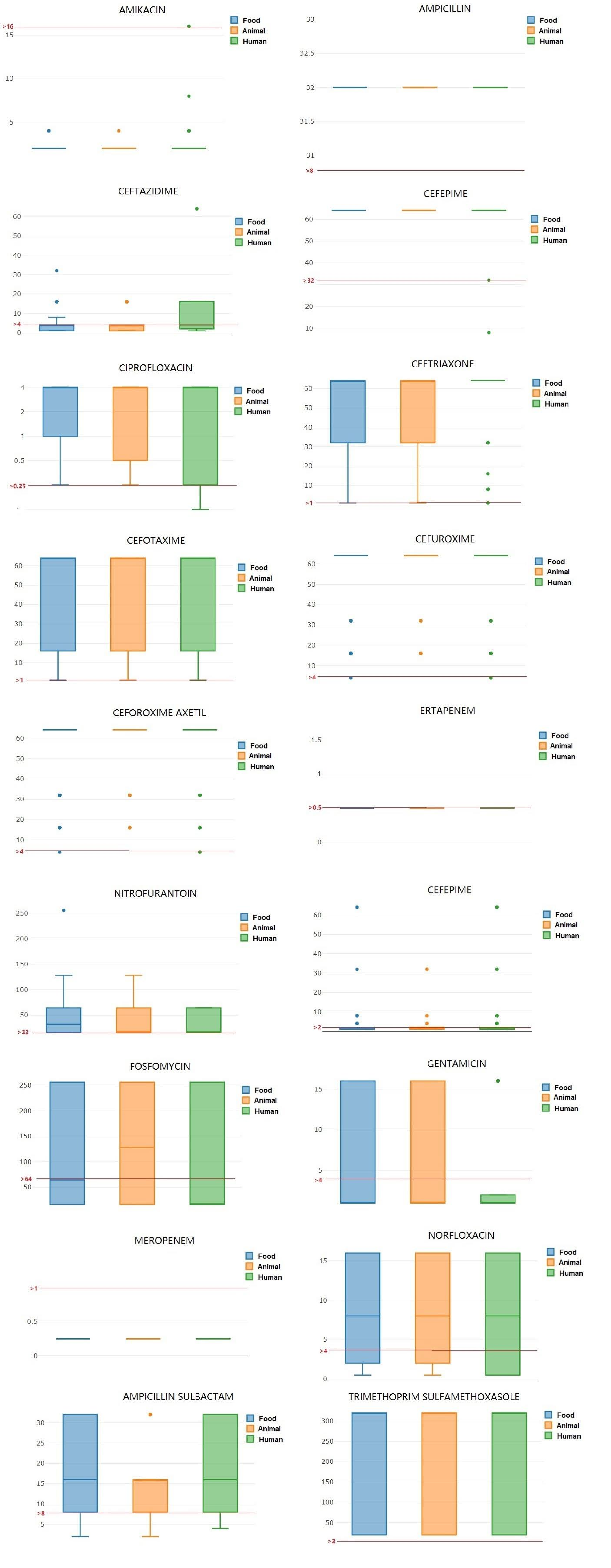

Supplement: Supplementary File 4 — Distribution of MIC values. [file Data_Sheet_1.PDF]
